# Commissioning of a motion management system for a 1.5T Elekta Unity MR‐Linac: A single institution experience

**DOI:** 10.1002/acm2.70005

**Published:** 2025-02-16

**Authors:** Blake R Smith, Joel St‐Aubin, Daniel E. Hyer

**Affiliations:** ^1^ Department of Radiation Oncology University of Iowa Iowa City Iowa USA

**Keywords:** commissioning, gating, motion management, MR‐Linac, unity

## Abstract

**Purpose:**

This work describes a single institution experience of commissioning a real‐time target tracking and beam control system, known as comprehensive motion management, for a 1.5 T Elekta MR‐Linac.

**Methods:**

Anatomical tracking and radiation beam control were tested using the MRI^4D^ Quasar motion phantom. Multiple respiratory breathing traces were modeled across a range of realistic regular and irregular breathing patterns ranging between 10 and 18 breaths per minute. Each of the breathing traces was used to characterize the anatomical position monitoring (APM) accuracy, and beam latency, and to quantify the dosimetric impact of both parameters during a respiratory‐gated delivery using EBT3 film dosimetry. Additional commissioning tasks were performed to verify the dosimetric constancy during beam gating and to expand our existing quality assurance program.

**Results:**

It was determined that APM correctly predicted the 3D position of a dynamically moving tracking target to within 1.5 mm for 95% of the imaging frames with no deviation exceeding 2 mm. Among the breathing traces investigated, the mean latency ranged between −21.7 and 7.9 ms with 95% of all observed latencies within 188.3 ms. No discernable differences were observed in the relative profiles or cumulative output for a gated beam relative to an ungated beam with minimal dosimetric impact observed due to system latency. Measured dose profiles for all gated scenarios retained a gamma pass rate of 97% or higher for a 3%/2 mm criteria relative to a theoretical gated dose profile without latency or tracking inaccuracies.

**Conclusion:**

MRI‐guided target tracking and automated beam delivery control were successfully commissioned for the Elekta Unity MR‐Linac. These gating features were shown to be highly accurate with an effectively small beam latency for a range of regular and irregular respiratory breathing traces.

## 1 INTRODUCTION

Motion management has played a critical role in modern external beam radiotherapy for the treatment of abdominal and thoracic tumors as an effective healthy tissue‐sparing treatment technique. Modern applications of respiratory gating focus the treatment on a smaller volume by only treating over a fraction of the patient's respiratory cycle that confines the motion of the target to an acceptable range. Consequently, accurate tracking and localization of the target is critical for accurate treatment delivery and satisfactory clinical outcomes. To‐date, several methods have been clinically investigated to localize, track, or verify the intrafraction motion of a target or surrogate including 4DCT,^1^ fluoroscopic imaging with and without radiopaque markers,^2^ surface guidance,^3^ ultrasound,^4^ and cine MRI.^5^, ^6^, ^7^

The inclusion of MRI for adaptive radiotherapy offers several advantages to other techniques, the most notable being the ability to continuously image with excellent soft tissue contrast and a lack of imaging dose. Since its introduction, numerous clinical applications have explored the use of MRI for cardiac monitoring,^8^ lung SBRT,^6^ and spine SBRT.^9^ To achieve the necessary real‐time monitoring, repeat imaging or cine monitoring is employed by multiple vendors to monitor the anatomical alignment during the treatment, directly monitoring the intended target rather than relying on a set of motion surrogates that may not correlate to the motion of the intended target.^10^ For these reasons, the integration of real‐time anatomy tracking and beam control in MRI‐guided adaptive radiotherapy (MRIgART) has been the focus of many recent investigations. An early application of anatomical tracking and automatic beam control was introduced by ViewRay for their MR‐Linac,^7^ which initially utilized three physical Co‐60 sources for treatment resulting in a prolonged beam latency. A recent publication from Charters et al.^5^ describes a dosimetric evaluation of MR‐guided respiratory gating and investigated how cine frame rate, latency, and gating window affect the resultant dose distribution for the ViewRay MR‐Linac, known as the MRIdian. Recently, Elekta has released their own comprehensive motion management (CMM) system to enable intrafraction target tracking and beam control with promising clinical results.^11^ Given the importance of characterizing these active monitoring and gating features to best facilitate their clinical use and understand their limitations, it was the purpose of this work to present the first clinical commissioning in North America for the Elekta Unity CMM system for MR‐guided anatomy tracking and beam gating.

## 2 METHODS

### 2.1 MRIgART system description and the traditional clinical workflow

Motion monitoring and real‐time gated treatment delivery was recently enabled for the Elekta (Stockholm, Sweden) Unity MR‐Linac. The Unity system is a commercially available MRI‐guided adaptive radiotherapy (MRIgART) platform that consists of a 1.5 T Philips 70 cm big bore MRI (Philips Healthcare, Best, Netherlands) combined with a standing‐wave, 7 MV flattening filter free (FFF) radiation beam. Intensity modulation is achieved along the in‐plane Supplementary Material) direction using a step‐and‐shoot technique with an Agility 160 leaf multileaf collimator (MLC). The accelerator and stationary Agility head are mounted to a slip‐ring gantry with a source‐to‐central axis distance of 143.5 cm and the system is capable of IMRT treatment deliveries across a deliverable treatment field of 57.4 cm cross‐plane (lateral direction) and 22.0 cm in‐plane (Supplementary Material direction).

Online adaptive planning is performed for each fraction that the patient is treated using either the adapt‐to‐position (ATP) or adapt‐to‐shape (ATS) workflow.^12^ Both processes utilize a reference plan that is generated from a reference dataset that provides bulk‐density tissue assignments and establishes a robust planning template, optimization parameters, and set of treatment goals that are utilized for each treatment fraction and can be tailored to specific sites.^13^ Adaptive treatments are based on a daily acquired MRI that is registered to the reference planning dataset. ATP adaptive treatments utilize a rigid transformation for this initial alignment, preserving the anatomy that was used to generate the reference plan. This workflow effectively simulates a systematic patient shift in the initial alignment and the user has an option to utilize a segment aperture morphing algorithm and warm‐start beamlet weighting optimization to reproduce the original dose‐volume histogram (DVH) target coverage. Alternatively, a new treatment plan can be tailored uniquely to the patient's daily anatomy by deforming the reference plan's contours on the daily MR image and performing an optimization based on the original objectives (ATS). Due to the additional time required by the ATS workflow, an additional 3D MRI of the patient can be obtained to verify constancy of the target location and contours of nearby organs at risk to their respective contours set from the daily planning MR image. With CMM, anatomical motion is monitored in two planes (sagittal and coronal) during treatment using a live 2D MR cine balanced turbo field echo (bTFE) imaging sequence. The bTFE sequence consists of a 1.9 ms TE, 3.8 ms TR, a flip angle of 40 degrees a 1.13 mm × 1.13 mm pixel resolution, a 5 mm slice thickness, and a temporal resolution of 200 ms.^14^ Before the release of CMM, gating or terminating the beam has been done manually based on the user's discretion from the cine imaging.

### 2.2 Anatomical position monitoring, beam control, and intrafraction plan adaptation

The comprehensive monition management (CMM) system provides multiple options for automatic gating of the radiation beam during treatment. A non‐respiratory gating technique referred to as *exclusion gating* in CMM will automatically pause the beam in the event that the target is detected to have moved outside of a user defined threshold in the bTFE image. The tracking of the target from the bTFE cine MR images is accomplished via the anatomical positioning monitoring (APM) system. The non‐respiratory gating technique has no limitation on the 3D pre‐treatment imaging that can be used for adaptive planning. Respiratory gating techniques within CMM include *expiration gating*, *average gating*, and *breath hold*. Respiratory gating within the CMM includes an algorithm to predict target motion that mitigates the latency caused by image acquisition and reconstruction, data processing, and beam on/off delays. Respiratory gating techniques are currently tied to specific 3D MR imaging sequences used for adaptive planning. For example, the *expiration gating* requires the use of the T2 navigator triggered imaging sequence, which acquires images at the exhale breathing phase. The *average gating* technique requires the use of a balanced or T1‐weighted 3DVane sequence. The 3DVane sequences consist of a repetitive radial acquisition of k‐space that creates a motion‐averaged MRI.^11^, ^15^ Lastly, breath hold sequences (balanced, T1, and T2) are required when using the *breath hold* gating technique. These restrictions are placed in order to ensure that the appropriate imaging is used for the gating strategy selected. In cases where the target is not well visualized on a bFTE cine MR image, the user can use a surrogate structure for motion tracking. The motion of the target with respect to the surrogate structure is assumed to be fixed, so care must be used in the selection of an appropriate surrogate structure. Specific MRI acquisition parameters are listed in Table 1.

**TABLE 1:** MRI parameters for a subset of 3D image sequences that are used to perform daily plan adaption with respiratory gating techniques. The pulse repetition time (TR), echo time (TE), scan duration, flip angle, acquired voxel size and thickness (thick), and field of view (FOV) along the lateral (Lat), vertical (Vert), and longitudinal (long) directions.

	Duration (ms)		Duration	Flip	Voxel (mm)		FOV (mm)		
Sequence	TR	TE	(mm:ss)	angle	size	thick	Lat	Vert	Long
Abdomen b3DVaneXD	3.3	1.31	4:07	40	1.74	3.00	500	500.0	220.5
Thorax b3DVaneXD	3.3	1.31	3:54	40	1.74	3.00	500	500.0	220.5
Abdomen T1 3DVaneXD	3.9	1.18	6:00	15	1.74	3.00	500	500.0	220.5
Thorax T1 3DVaneXD	3.9	1.18	6:00	10	1.74	3.00	500	500.0	220.5
Abdomen T1 3D BH	6.2	4.0	0:17	10	2.00/2.01	4.00	554	320.5	280.0
Thorax T1 3D BH	6.2	4.0	0:17	10	2.00/2.01	4.00	554	320.5	280.0
Abdomen T2 3D BH	1200	166	0:18	90	2.20/2.51	5.00	360	484.5	280.0
Thorax T2 3D BH	1200	166	0:18	90	2.20/2.51	5.00	360	484.5	280.0

Once the adapted plan is created for any respiratory technique, the CMM acquires a template image in order to create a registration between the 2D bTFE cine MRI images and the 3D MRI acquired for adaptive planning. Subsequent tracking of the target in APM is accomplished by comparing the real‐time cine bTFE images with the template images. Registration of the template images to the 3D MRI allows for the quantification of any motion immediately before tracking begins. The template is generated by acquiring a 60‐frame cine divided equally between the coronal and sagittal planes that are centered on the tracked target location.

When performing a gated delivery, the beam is gated when the volumetric overlap of the target and the gating envelope drops below a user‐defined limit (e.g., 95%) or if the motion exceeds a defined displacement threshold in either the *X*, *Y*, or *Z* directions.

### 2.3 Experimental setups

**FIGURE 1:**
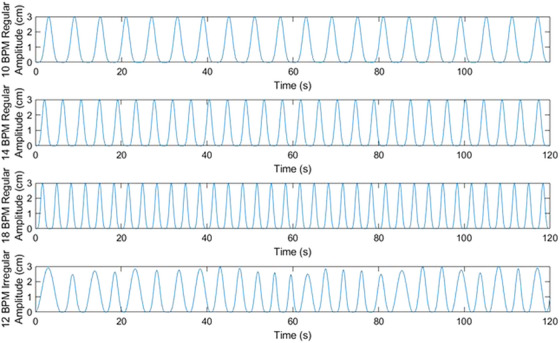
Two‐minute time series of the breathing traces utilized for this work. Regular, consistent breathing patterns were modeled using a cos^6^ function for 10, 14, and 18 BPM breathing rates. A more realistic breathing trace was investigated at 12 BPM utilizing a variable amplitude, period, and waveform. BPM, breaths per minute.

Commissioning and validation of the Unity CMM system was performed on a clinical MR‐Linac. Specific details regarding the commissioning efforts have been previously published^16^ and consist of dosimetric calibration and characterization, IMRT treatment delivery validation, baseline characterization of MR imaging capabilities, and imaging coincidence with the treatment beam. The CMM system entails numerous unique features that build upon the existing Unity treatment framework to enable gated treatments based on the MR cine images. In order to test this system, a set of four breathing traces was developed to simulate a range of realistic breathing patterns with the MRI^4D^ QUASAR phantom (MODUS Medical Devices, London, Ontario). The traces are shown in Figure 1 and are included as Supplemental Material to this manuscript. Three standard breathing traces at 10, 14, and 18 breaths per minute (BPM) were modeled using a $\mathrm{co}s^{6}$ function with a 30 mm amplitude. A moderately irregular breathing trace was also developed to evaluate the gating system using a simulated breathing trace modeled after a realistic, self‐paced breathing cycle. This was accomplished by using a random combination of $\mathrm{co}s^{2}$, $\mathrm{co}s^{4}$, and $\mathrm{co}s^{6}$ functions with a uniformly distributed period ranging between 10 and 14 BPM (mean of 12 BPM) and a uniformly distributed amplitude ranging between 26 and 30 mm (mean of 28 mm). A MnCl_2_ contrast solution was used for the target, surrounding insert, and phantom body at concentrations specified by the manufacturer, MODUS Medical Devices.^17^

**FIGURE 2:**
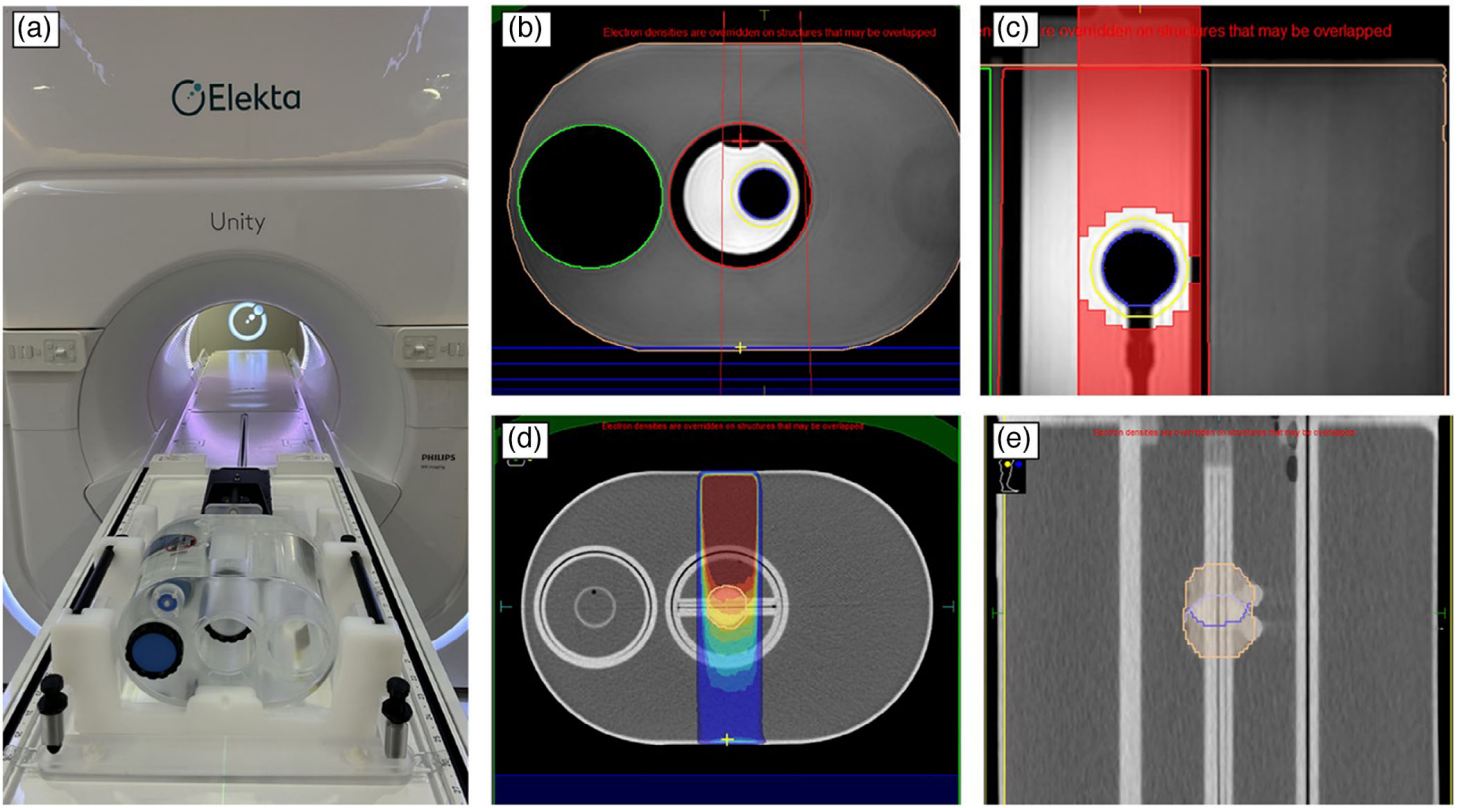
(a) Experimental setup of the MRI4D Modus respiratory phantom and cradle. (b) Static treatment fields, shown from the red vertical field lines, were planned to use the off‐center target with a single collimated treatment shown by the red MLC projection. (c) This fixed field from a static target position was used to generate a treatment delivery to test the APM and beam latency accuracy. (d) A separate, single‐field treatment plan was generated using a centrally located target with a 30 mm motion amplitude that contained a film insert module, which was (e) gated based on an ITV target created from the target positioned at 0 and 15 mm. APM, anatomical position monitoring; MLC, multileaf collimator.

During testing, the physical motion of the phantom was recorded for the *X* (lateral), *Y* (vertical), and *Z* (longitudinal) directions from the encoder simultaneously with the beam‐control signal that was synchronized to the same digital clock on the Unity data processing PC (DPPC) using the Modus pResp Quasar software (Version 4.2.12). This computer is used for a variety of QA activities and is part of the Unity server rack. Any device or software application that operates directly from the DPPC will inherently share the same digital clock with the computer that operates the Linac as it is synced to the same time server. This software contains specific applications for the CMM testing such as APM accuracy and latency, which are described in Sections 2.3.1 and 2.3.2, respectively. Two treatment plans were created to evaluate the APM predictive accuracy and beam gating latency as well as quantify the dosimetric impact of the system latency. Figure 2 illustrates the treatment planning setup that consisted of a single field at a gantry angle of 0 degrees directed toward the MODUS phantom. In the first treatment planning case, an off‐centered target was placed into the motion stage of the MODUS phantom and imaged at a stationary position of 0 mm on the Modus software. To evaluate APM latency, a PTV expansion of 5 mm around the target was used, and the gating window was set equal to the PTV. A fixed MLC field was then set to match the PTV with no additional blocking margin. A dosimetric study was later performed in which the beam was gated during a portion of the target's total 30 mm of motion. The ITV was defined by a volume that encompassed the target's amplitude between 0 and 15 mm. In this dosimetric gating study, a PTV expansion of 5 mm around the ITV was used and again the gating window was set to the PTV with no additional MLC block margin. The respiratory gating technique was used for all APM investigations.

#### 2.3.1 APM predictive accuracy

The error in the predicted motion of a target was benchmarked for each of the four breathing traces shown in Figure 1 following an online ATP workflow. The daily 3D MR image was obtained with no motion while a small (0.2 mm at 15 BPM) amount of motion was initiated on the MODUS phantom to enable the template acquisition. Following the template acquisition, the breathing traces shown in Figure 1 were played on the MODUS phantom. Motion in three directions was enabled by engaging the rotational arm on the translational stage, which induced a ±30 degree rotation across the entire longitudinal range of motion of the translational stage. To induce motion in the *X* and *Y* directions of the target, a base insert for the tracking target was offset by 1.5 cm to induce a preset oscillatory motion among all three directions that is synchronized with the translational stage and has a known positional relationship that is calculated within the MODUS software. The APM system monitored the motion trace continuously for at least 5 min prior to terminating the session. The tracking history log is extracted from the Unity DPPC and analyzed against the MODUS motion trace for each synchronized time point to evaluate the accuracy of the APM predictive accuracy against the actual known position from the MODUS phantom.

#### 2.3.2 System latency

Beam gating latency was initiated following the same ATP and template acquisition workflow used to assess the APM predictive accuracy. To gate the beam, a displacement threshold technique was enabled. This method gates the beam when the target position is outside the defined bounds, which were set at 5 mm in the superior and inferior direction of motion. The analog input of the latency module of the MODUS control system was set to sample at 2 kHz without digital filtering and connected to the Linac signal.^17^ Deliveries were run to obtain satisfactory statistics (minimally 100 sampled instances of beam gating) for each simulated breathing trace.

#### 2.3.3 Dosimetric beam constancy

Beam output and profile constancy were verified for several gating windows enabled using a gating applet in Service mode that was installed at the time of an upgrade to the Unity system in preparation for gating. The applet supplies a gating signal directly to the Linac control, bypassing the imaging and CMM systems. This is necessary given that the gating capabilities from CMM rely solely on a real‐time MR signal and cannot otherwise be bypassed. Using the applet, a subset of gating windows was created and consisted of a lightly gated sequence consisting of alternating 2 s beam‐on and 1 s beam‐off windows, a moderate gating sequence of alternating 0.5 s beam‐on and 1 s beam‐off windows, and a highly gated sequence of alternating 0.05 s beam‐on and beam‐off windows. The dosimetric constancy during beam gating was evaluated for output, in‐plane, and cross‐plane beam profiles using an MR IC Profiler (Sun Nuclear, Melbourne, FL, USA), which was compared to an ungated beam delivery. Each delivery utilized a 20 cm × 20 cm field and a 100 MU output delivery and was compared relative to an ungated beam delivery.

#### 2.3.4 Dosimetric impact of gating latency

The dosimetric impact of the total system latency and predictive error was evaluated for gated, fixed‐field treatment delivery. For this assessment, an ideal gated delivery with no latency was modeled by convolving a static field delivery measured on Gafchromic film (Ashland, Bridgewater NJ, USA) with a known motion trace. In this ideal scenario, where a treatment could be delivered without any latency and perfect APM accuracy, the resultant dose distribution was modeled as a static‐field dose profile whose convolution reflects the positional displacement of the motion stage within the specified gating window. This ideal delivery was then compared to an actual delivery on Unity using CMM and the same motion trace used in the convolution to investigate the dosimetric impact of gating latency. To model the reference profile with no latency, $D_{\mathrm{ref}}$, a static field, $D_{\mathrm{static}}$, was measured and convolved from the known positional displacements recorded from the MODUS phantom throughout each detected time span, $\delta t_{i}$, of the total beam delivery, $\sum_{i=1}^{N}\delta t_{i}$, of N segments. The spatial range confining the convolution was set as the expected gating window; although each breathing trace consisted of a 30 mm amplitude, the beam was gated within the range of 0 to 15 mm. Specifically for this analysis, the resultant dose profile was calculated using Equation (1),

(1) $$D_{\mathrm{ref}}\;\left(x,z\right)=\frac{\sum_{i=1}^{N}D_{\mathrm{static}}\left(x_{0},z_{0}+\Delta z\left(t_{i}\right)\right)\cdot \delta t_{i}}{\sum_{i=1}^{N}\delta t_{i}}$$

where each time point was considered sufficiently small and of equal contribution to the resultant dose profile. Planar 2D dosimetry was performed with Gafchromic EBT3 film (LOT# 09072202) using a specialized insert containing a film holder that was positioned between the two halves of the target structure as shown in Figure 2. The film was calibrated using a 6 MV calibration curve. Although the Unity MRLinac utilizes a 7 FFF beam quality, the energy response of Gafchromic film has been shown to be negligible among clinical MV beam energies ranging between 6 MV and 15 MV.^18^ No additional corrections were applied for the presence of the 1.5 T magnetic field due to the small dose‐response shown for Gafchromic film in the presence of magnetic fields.^19^ Additionally, EBT3 films were strictly used for only relative comparisons in this work.

Dosimetric convolution and analysis were performed using a set of custom scripts written and executed in MATLAB (Version 2020b, Natick, MA, USA). Each measured dose profile was analyzed using the red color channel and underwent median filtering. Point‐based registration was used to register the analytically convolved and measured dose distributions from a set of pinprick fiduciary marks on the film from the film holder insert. The dosimetric impact of the system latency for each breathing trace was quantified using a series of global gamma analyses^20^ using a 10% low‐dose threshold with gamma criteria of 3%/2 mm, 2%/2 mm, and 1%/1 mm, which compared the measured gated dose profile against the analytically convolved dose distribution that was set as the reference dose profile.

## 3 RESULTS

### 3.1 APM predictive accuracy

**FIGURE 3:**
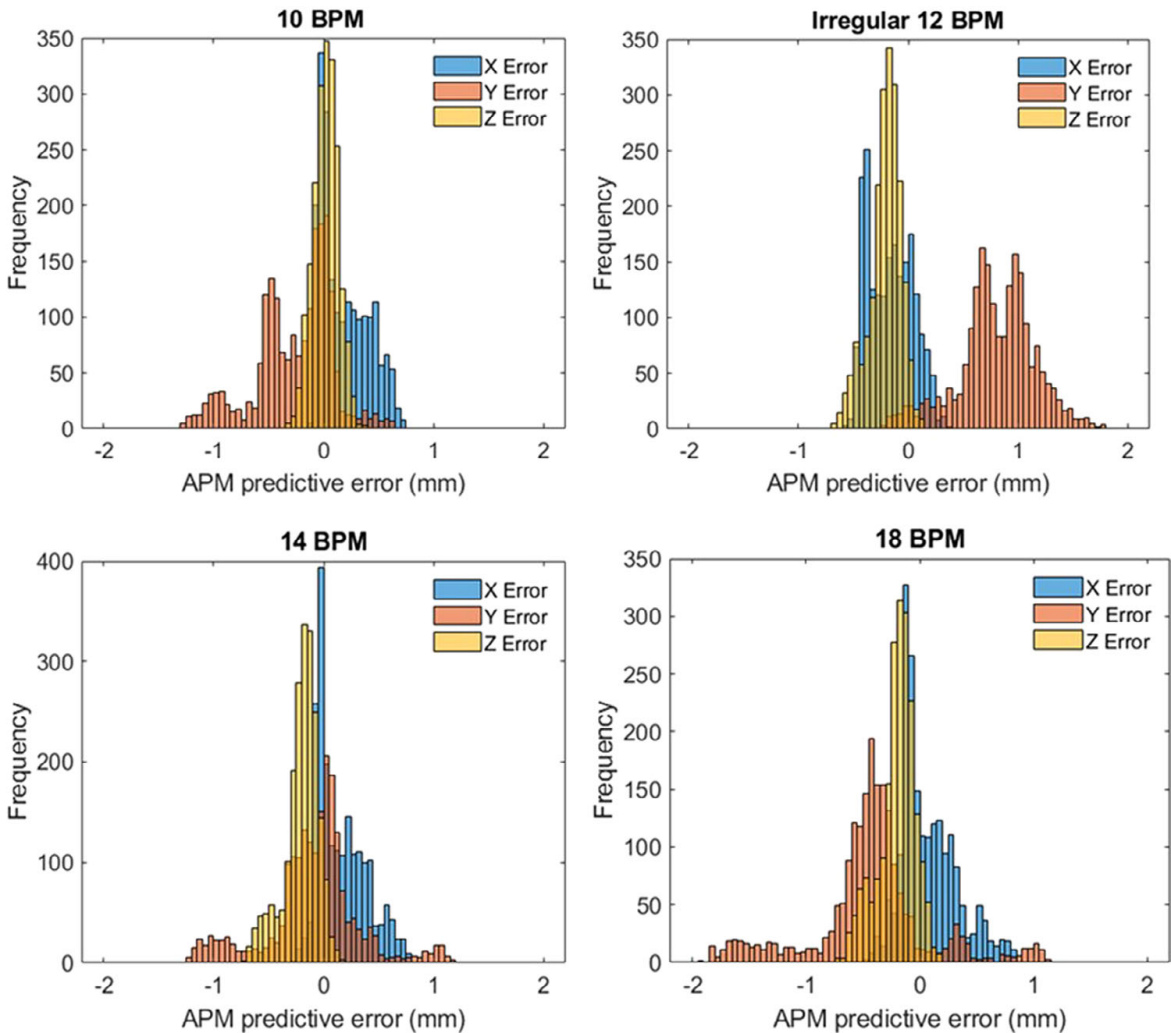
Distribution of the predicted positioning error along each primary axis of motion from the anatomical position monitoring (APM) system for each of the breathing traces shown in Figure 1.

The APM predictive error was evaluated for all planes of motion caused by the QUASAR off‐center target piston insert among the four breathing traces. Figure 3 shows the histogram of calculated APM errors between the algorithm's predicted target displacement and the known translation from the MODUS QUASAR phantom. Table 2 lists the statistical mean, standard deviation, and 95th percentile APM errors and their associated vector magnitude. Additionally, the percentage of predictive values that were below a threshold of 1 and 2 mm were determined for each breathing trace and are listed in additional columns. The final predictive error results listed in Table 2 have omitted any instances where the APM system detected poor tracking accuracy and would have prevented beam delivery; errors were only considered in this analysis if the decision to treat was instantiated by the APM system. For example, low quality and low accuracy inhibits are present at start of APM. This is the expected behavior of the system to inhibit treatment while the system prepares and validates its predictive model. There were no tracking errors or inhibits outside of this initial preparation stage for the constant 10 and 14 BPM breathing cycles. Roughly 11% of the time there were tracking inhibits for the irregular breathing trace. Under extreme circumstances, 25% the 18 BPM breathing trace with a 30 mm amplitude consisted of tracking inhibits. While 25% represents a large fraction of treatment inhibits, the 18 BPM breathing trace with a 30 mm motion amplitude is an extreme scenario not likely to be encountered frequently for clinical cases.

**TABLE 2:** Anatomical positioning monitoring (APM) statical mean, standard deviation, and 95th percentile of the error along each primary axis and net magnitude vector for the breathing traces shown in Figure 1. The relative abundance of predicted target positions relative to their known location was calculated for a 1 and 2 mm threshold.

		APM predictive error (mm)			Percent (%) within	
Trace	Direction	Average	Stdev	P95%	1 mm	2 mm
10BPM	*X*	0.2	0.2	0.6	100.0	100.0
	*Y*	0.3	0.3	1.0	94.5	100.0
	*Z*	0.1	0.1	0.2	100.0	100.0
	Vector	0.4	0.3	1.1	92.2	100.0
14BPM	*X*	0.2	0.2	0.6	100.0	100.0
	*Y*	0.3	0.3	1.0	92.7	100.0
	*Z*	0.2	0.2	0.6	100.0	100.0
	Vector	0.5	0.3	1.1	91.1	100.0
18BPM	*X*	0.2	0.2	0.6	100.0	100.0
	*Y*	0.6	0.4	1.5	85.7	100.0
	*Z*	0.2	0.2	0.5	100.0	100.0
	Vector	0.7	0.4	1.5	81.6	100.0
12BPM irregular	*X*	0.2	0.1	0.4	100.0	100.0
	*Y*	0.8	0.3	1.4	71.4	100.0
	*Z*	0.2	0.1	0.5	100.0	100.0
	Vector	0.9	0.3	1.4	56.9	100.0

### 3.2 System latency

**FIGURE 4:**
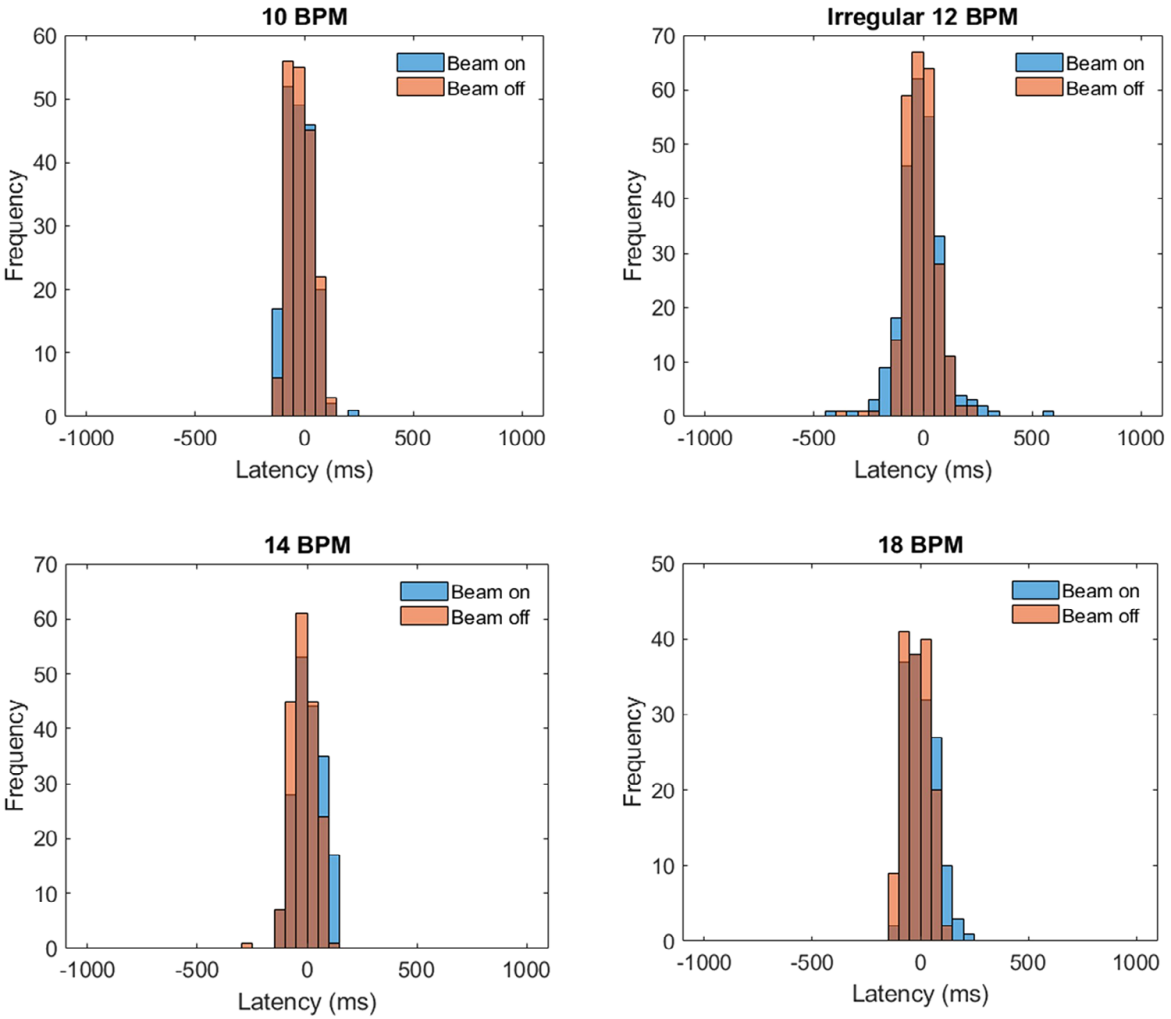
Distribution of the beam latency from a gated treatment delivery using the anatomical position monitoring (APM) system. Beam‐on (blue) and beam‐off (red) latencies were evaluated for each of the breathing traces shown in Figure 1.

The beam‐on and beam‐off latency was obtained for the four breathing traces shown in Figure 1. Each of these traces was further segmented into the positive and negative latency components that resulted in the portion of the latency that is associated with a delayed (late) or prematurely (early) gating response. These results are plotted in the histograms shown in Figure 4 with the associated statistical analysis results for each distribution listed in Table 3. A beam delivery inhibits otherwise prevent the system from gating, which is present during the first few seconds of tracking while the APM algorithm is learning. The latency values presented in Table 3 were calculated based on the magnitude of latency where the column listed as Total reflects the composite of both early and late latency components. The one‐sided bound of the statistical latency range containing 95% of all latency observations is listed in Table 3 along with the portion of measured latencies that were within 100 and 200 ms.

**TABLE 3:** Beam‐on and beam‐off latency statical means including both the positive and negative (standard deviation, and 95th percentile relative abundance beam latencies that are within a 100 and 200 ms threshold.

	Breathing	Beam latency (ms)						P95%	Percent (%) within	
	trace	Average		Left‐sided (negative)		Right‐sided (positive)		(ms)	100 ms	200 ms
Beam‐on	10 BPM	−21.7	(60.4)	−41.8	(38.0)	58.9	(34.6)	110.5	87	99.7
	14 BPM	7.9	(63.5)	−58.0	(38.7)	46.3	(32.8)	117.1	83.9	100
	18 BPM	6.3	(67.8)	−63.3	(48.2)	47.8	(26.9)	124.8	86.9	99.1
	12 BPM	−11.0	(98.7)	−67.0	(79.1)	72.3	(62.9)	188.3	71.6	93.5
Beam‐off	10 BPM	−16.9	(54.9)	−42.7	(28.4)	52.2	(30.8)	99.2	94.8	100
	14 BPM	−18.2	(55.7)	−38.0	(27.2)	52.7	(37.8)	99.8	94.1	99.6
	18 BPM	−17.0	(58.1)	−41.4	(31.0)	58.2	(31.1)	105.5	90.8	100
	12 BPM	−11.3	(71.1)	−50.5	(45.4)	57.1	(48.1)	130	81.8	97.2

*Note*: Both negative (pre‐emptive latency) and positive (delayed) beam latency are obtained for each of the breathing traces shown in Figure 1.

### 3.3 Dosimetric beam constancy

**FIGURE 5:**
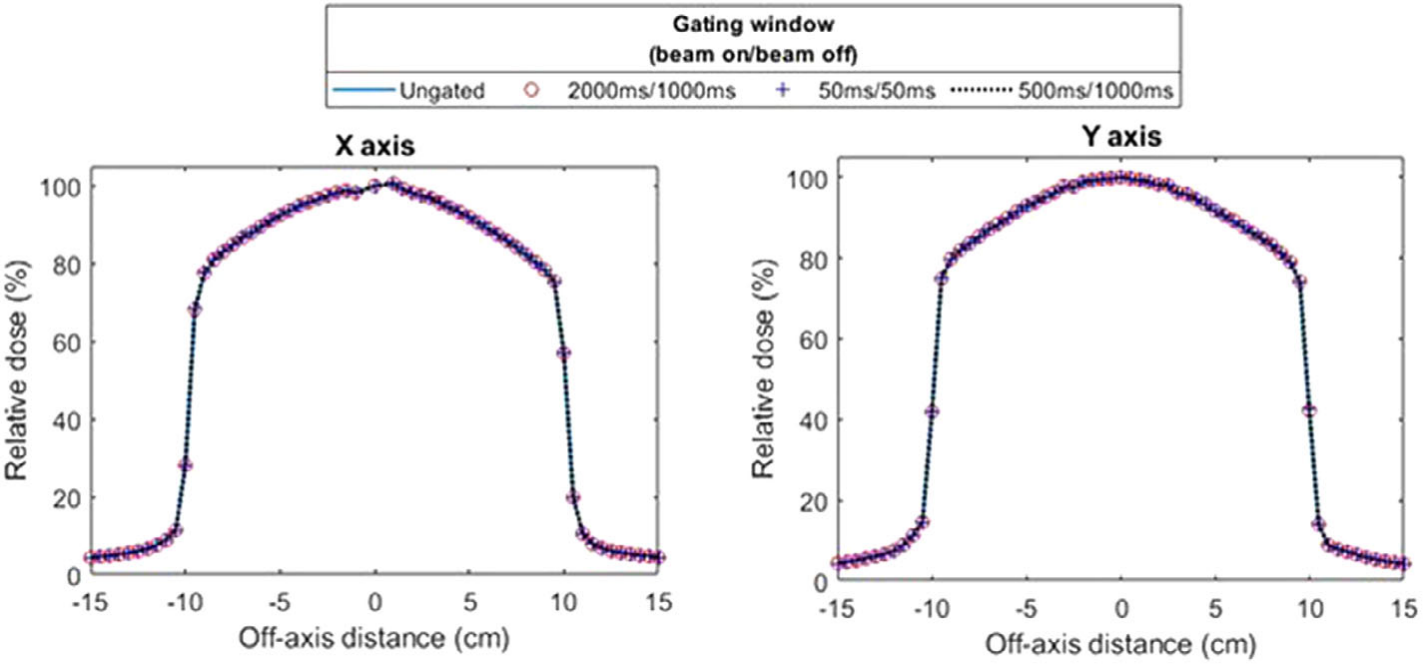
Cross‐plane (*X*‐axis) and in‐plane (*Y*‐axis) dose profiles measured with the MR IC‐Profiler for multiple gating windows.

The in‐plane and cross‐plane profiles measured using the MR IC Profiler for three different gating windows are shown in Figure 5. Great agreement was observed among each profile, where they were found to coincide within the reproducibility of the measurement with an ungated beam delivery. Table 4 lists the profile features measured from the IC Profiler software.

**TABLE 4:** Dose profile metrics taken from the MR IC‐profiler software relative to an ungated treatment field for the central axis dose (CAX), field size (FS), lateral penumbra defined as the 80% to 20% width, field flatness, and symmetry.

		*X*‐axis				*Y*‐axis			
Gating Window	CAX (%)	FS (cm)	Penumbra (cm)	Flatness (%)	Symmetry (%)	FS (cm)	Penumbra (cm)	Flatness (%)	Symmetry (%)
50 ms On / 50 ms Off	−0.06	0.01	−0.02	−0.2	0	0.01	0.01	−0.1	0
500 ms On / 1 s Off	−0.15	0.01	−0.03	−0.3	0	0.01	−0.03	−0.1	0.1
2 s On / 1 s Off	−0.18	0	−0.02	−0.2	0	0	−0.01	−0.1	0

### 3.4 Dosimetric impact of gating latency

**FIGURE 6:**
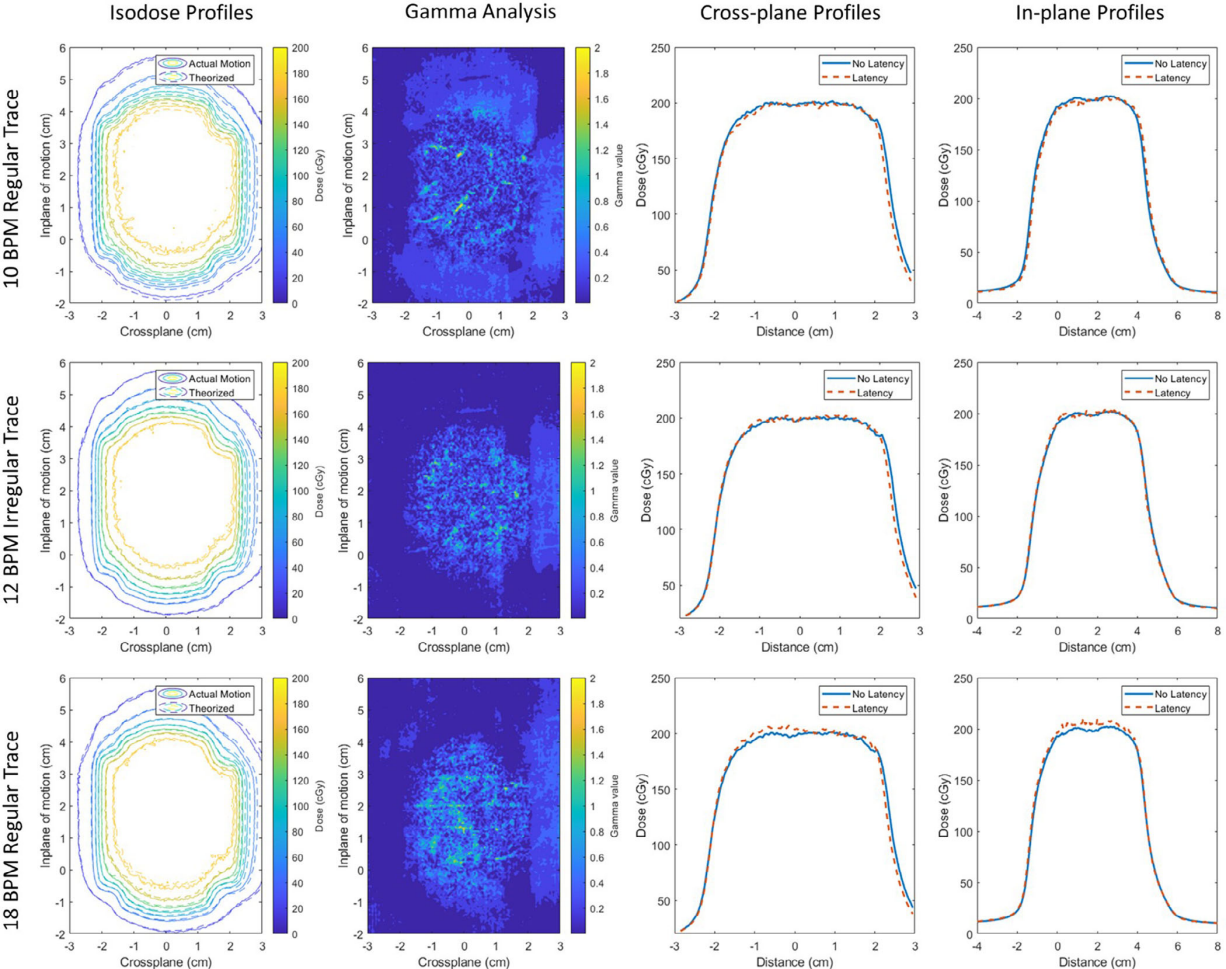
Dose distribution and gamma analysis plots for a selection of breathing traces illustrated in Figure 1. The dosimetric impact of system latency was evaluate for measured gated dose distributions (shown with dashed lines) against a theoretically determined gated dose profile delivered without latency (solid line). The 3%/2 mm gamma analysis is shown for each breathing trace comparison. Cross‐plane and in‐plane profiles are shown for both dose profiles obtained across a small band centered at 2 and 0 cm, respectively.

The dosimetric impact of latency and tracking accuracy was characterized by the similarity of the measured profile to a theoretical dose distribution that would have been delivered without the presence of these factors. Figure 6 shows the set of dose distribution comparisons between the measured dose profiles that included system latency against their analytical counterparts. The system latency that was observed in this work appears to have a minimal impact on the resultant dose distribution, which is summarized in Table 5. The Gamma analyses showed that all 2D dose profiles match their analytical counterpart above 97% for a 3%/2 mm criterion and above 93% for a 2%/2 mm criterion. Additionally, an average dose from the 2D dose distribution was sampled within the central, high‐dose, and low‐gradient regions to quantify the potential difference in the delivered output. Sub‐percent differences for breathing rates less than 12 BPM were observed with the highest dose difference of 1.8% observed for the fastest breathing rate of 18 BPM. The dose differences were within the expected uncertainty of the film's calibration and inter‐film variability (Table 5).

**TABLE 5:** Dose distribution comparisons quantifying the impact of latency on gated treatment deliveries against an analytically modeled gated treatment delivery without latency for a selection of breathing traces illustrated in Figure 1.

		Gamma pass rates (%)		
Breathing trace	Dose diff (%)	3%/2 mm	2%/2 mm	1%/1 mm
10 BPM regular	0.2	98.4	96.9	83.3
12 BPM irregular	0.6	98.3	96.1	86.6
18 BPM regular	1.8	97.3	93.3	81.9

*Note*: Dose difference was evaluated over a small region of interest manually centered in the high dose, low‐gradient region of the dose profile. Global gamma analysis tests were evaluated for points above a 10% threshold limit.

## 4 DISCUSSION

### 4.1 CMM gating commissioning and comparison to other literature

A unique feature of the APM system is its ability to mitigate the total system latency through a predictive algorithm that compensates for the time to acquire and reconstruct an image (200 ms or 5 frames per second), data processing, and any delay in triggering the beam off and on. Without this feature, the treatment delivery would not compensate for this delay resulting in a persistent latency behind the triggered signal from the APM system. This is not expected to have a significant dosimetric impact if the beam is infrequently gated, such as in the scenario of exception gating where the beam is paused only if the target migrates outside the intended treatment region but is otherwise expected to remain within the targeted region throughout the length of the treatment. Latency compensation is otherwise enabled for respiratory techniques including respiratory, exhale, and breath‐hold techniques, and is an essential feature to improve the delivery accuracy and treatment efficiency. As such, the central focus of this commissioning work specifically characterizes the impact of the system accuracy with respect to target tracking and predictive modeling using these respiratory techniques. This is of particular dosimetric interest as the system can, and does, result in both positive and negative latency as a result of the predictive error. As shown in the histogram plots of Figure 4, the average latency is near zero, and more than 99% of all observed latencies were within 200 ms across the range of regular simulated breathing traces, which is well within the AAPM guidelines for targeted system latency for radiotherapy gating.^21^ The uncertainty of the APM error (difference) measurements in Table 1 is influenced by the precision of the MODUS motion stage and the resolution of the APM tracking system. The MODUS motion stage has a position precision of 0.25, 0.5, and 0.25 mm for the *x*, *y*, and *z* directions, respectively.^17^ Modeling the uncertainty of the APM tracking system as a rectangular function spanning the dimensions of a voxel,^22^ the *k* = 2 expanded uncertainty can be estimated for the APM predictive error as 1.40, 1.64, and 1.40 mm in the *x*, *y*, and *z* directions, respectively. In context to the results in Table 1, the average errors are within the *k* = 1 uncertainty of the measurement and the measured P95% predictive error values were within the *k* = 2 confidence for all breathing traces.

In comparison to other MR‐guided gating technologies, the results from this work are promising, showing how MRigART has matured into an effective modality for intrafraction gating and adaptive planning. Earlier works of similar technologies showed average latencies ranging between 246 and 527 ms during the infancy of respiratory‐gated MRIgART that utilized mechanical transitioning of Co‐60 sources.^7^ Similar studies quantifying latency of counterpart MR‐Linac gating systems have found that the average latency ranges between 128 and 243 ms if four planes‐per‐second imaging is used and between 47 and 302 ms when eight planes‐per‐second cine imaging is used.^23^ A similar dosimetric study evaluated seven realistic breathing traces, similar to the traces utilized in this work, and found that the overage beam‐off latency was 150.6 and 118.1 ms with 4 and 8 frames per second, respectively, and the average bean‐on latency was 318.1 and 597.4 ms with 5 and 8 frames per second, respectively.^5^

The dosimetric impact of the system latency, as well as the system latency itself, is dependent of the breathing trace and tracking algorithm. Similar works have performed dosimetric analyses of respiratory gating on MR‐guided respiratory gating and have observed a notable degradation against the reference static plan for shorter respiratory periods.^5^ Against a static reference plan, the gamma pass rate from the average measured gated dose distribution was between 9% and 80% lower. In comparison to this work, the gamma pass rates do not equally reflect the same comparison; the impact of motion on the resultant dose distribution is accounted for with our convolution technique following a time‐integrated approach.^24^ However, our results share common trends including a small degradation in the constancy of the delivered gated dose distribution as the breathing frequency increases where irregularity in the breathing trace appears to have a more significant effect on the resultant dose profile. However, in all cases, the gamma passing rates were above 97% for our institution's clinical gamma criterion of 3%/2 mm, which is above the recommended patient‐specific quality assurance threshold of 90% for a 3%/2 mm criterion.^25^ The averaged dose sampled in the high‐dose, low‐gradient region was within the uncertainty of the film calibration, which is a representative evaluation of the targeted GTV coverage based on the PTV and applied gating envelope. The inherent latency within the respiratory gating reduced the central dose for the regular breathing cycles, and the difference increased for the faster respiratory rates. Interestingly, while breathing irregularity appears to be the dominant factor that impacts the gamma comparison, breathing rate appears to impact the dose magnitude. From Table 2, we can see that the irregular breathing trace results in the largest beam‐on and beam‐off latency errors, which could intuitively affect the relative dose profile. However, this irregular breathing trace also has the highest variability of errors whereas the 18 BPM trace appears to have the second largest, but most consistent, latency errors, which may have contributed to the larger dose deviations observed within the central high‐dose region.

### 4.2 Workflow and technical consideration of adaptive baseline shift corrections

While there have been recently published recommendations on the clinical implementation of MR‐Linac systems in radiation oncology,^26^ these reports lack specific recommendations regarding MR‐guided anatomical tracking and beam gating. As such, the clinical integration of gating on the Unity MR‐Linac poses new workflow considerations and additional quality control checks outside of the core implemented adaptive, site‐specific clinical workflows at our institution.^13^ For example, in addition to the active gating capabilities afforded with CMM, intrafraction *baseline shifts* can also be employed to recenter the treatment delivery if the target migrates outside the gating window. During a baseline shift, the average target offsets are sent to the online Monaco treatment planning system, where only the target structure is rigidly moved within the daily MR planning dataset. A segment aperture morphing (SAM) algorithm is applied to adjust the MLCs, while retaining the original planned monitor units, to recalculate the target coverage to guide the clinical decision on whether to proceed with the baseline shift procedure or perform a completion plan. Several trials were performed to verify the functionality of this procedure. No specific dosimetric verification was performed, as it was determined that this new functionality was purely technical and utilized previously commissioned aspects of the Unity and Monaco treatment planning platform. Specifically, the baseline shift functionality utilized existing features including the TPS SAM algorithm and the Monte Carlo dose calculation engine previously commissioned for our beam model.^16^ Visual inspection of the shifted target structure in the daily planning MR, constancy of planned monitor units, data transfer accuracy and independent point dose calculation using an MR‐commissioned beam model of the Elekta Unity in RadCalc (LAP, Boynton Beach, FL, USA)^27^ were used to verify the accuracy in the updated treatment parameters. While the baseline shift algorithm technically generates a new treatment plan, no additional patient‐specific quality assurance was deemed to be necessary given the close similarity of the baseline shift treatment plan to its reference plan, which our institution has demonstrated to a high statistical confidence that any adaptive plan will have a consistent gamma passing rate relative to their reference plan.^28^ It should also be noted that there exist multiple variations of the original online adaptive workflow from Elekta.^12^ Various modifications have been adopted across different clinics, such as in‐house extensions or customized sequences to incorporate 4D‐MRI as part of the daily adaptive planning,^29^, ^30^ use of contrast agents to enhance imaging,^31^ development of customized imaging sequences,^32^ automated planning algorithms,^33^ and deep learning auto‐segmentation algorithms.^34^ Given the variability of these customized tools, it is not clear how compatible they will be with the intended installation of CMM and therefore it will be an important commissioning task of the institution to properly validate any deviations from the intended workflow.

### 4.3 Modifications to an existing quality assurance program

The addition of beam gating is a powerful tool to enhance radiotherapy treatment capabilities but is subject to additional quality assurance as summarized in APPM task group 142^35^ and the respiratory management considerations from AAPM TG 76.^21^ Additionally, new interlock and safety features were validated including delivery inhibits following image quality errors, loss of cine MR imaging, and recovery workflows following treatment termination. Intentionally inducing specific imaging‐related gating inhibits is difficult due to the sensitivity of these interlocks to the imaging environment, which may not be reproducible. However, simple tests can be done to verify their functional operation including performing a faux baseline shift into a region where the target is no longer visualized or drastically changing the motion trace while tracking, resulting in an imaging quality or tracking error that inhibits beam delivery. It should be noted that the APM and latency commissioning tasks were completed multiple times throughout acceptance, commissioning, and following the first several months of clinical use referencing the tolerances from the aforementioned task groups as the minimum acceptance machine performance. Among repeated measurements, subsequent gated output and profile flatness and symmetry remained within 0.1% of their open‐field counterparts while 95% of all APM deviations remained within 2 mm. As part of our quality assurance program, we have included gated beam profile output and constancy checks monthly and annually utilizing the Elekta gating service mode applet. Additionally, APM accuracy will be validated monthly while APM accuracy and system latency will be validated annually following both the TG 142^35^ and TG 147.^36^ Often, some form of beam gating is included as part of patient‐specific beam delivery quality assurance^37^ to evaluate the additional strain of a gated delivery on a dynamic treatment delivery. However, given that the treatments on Unity are strictly step‐and shoot IMRT, no additional patient‐specific gated QA was incorporated at this time given the existing quality control measures that have been added.

## 5 CONCLUSION

This work summarizes the first institutional commissioning experience of the Elekta CMM and MR‐guided beam gated delivery system for Unity in the United States. The system upgrade offers numerous new features to Unity users including four beam gating techniques and an additional intrafractional treatment planning technique to account for baseline drifts. Each of these systems was verified and their accuracy characterized prior to clinical use following vendor and existing AAPM task group recommendations. The system latency, as a result of predictive error, APM tracking accuracy, beam profile and output constancy, and the dosimetric impact of the system latency were characterize for a variety of clinically representative regular and irregular breathing traces. In each of these scenarios, the Elekta Unity MR‐guided beam gating system demonstrated accurate target positioning prediction with a low beam delivery latency that appears to have a minimal impact on the overall resulted gated dose distribution.

## AUTHOR CONTRIBUTIONS

Dr. Smith: Lead author of manuscript, physics data collection and analysis, corresponding author. Dr. St‐Aubin: Study design, co‐author of manuscript, physics expertise, data collection and review. Dr. Hyer: Study design, senior author of manuscript, physics expertise, data collection and review.

## CONFLICT OF INTEREST STATEMENT

Dr. Daniel Hyer reports a relationship with Elekta that includes: consulting or advisory, funding grants, and speaking and lecture fees. Dr. Joel St‐Aubin reports a relationship with Elekta that includes: funding grants and speaking and lecture fees. Dr. Blake Smith has nothing pertinent to disclose.

## Supporting information



Supporting Information

Supporting Information

Supporting Information

Supporting Information
